# Developing a genetic return of results service core

**DOI:** 10.1017/cts.2025.10197

**Published:** 2025-11-17

**Authors:** Jennifer A. McKenzie, Erin McRoy, Kevin M. Bowling, Jorge Luis Granadillo De Luque, Jessica Mozersky, Erin Linnenbringer, Dustin Baldridge, Jonathan W. Heusel, Julie A. Neidich, Amanda F. Cashen, Laura J. Bierut, Sarah M. Hartz, Christina A. Gurnett

**Affiliations:** Washington University Institute of Clinical and Translational Sciences, Washington University in St. Louis, St. Louis, MO, USA

**Keywords:** Return of results, research participants, service, genetics, genomics

## Abstract

Research participants should be informed of genetic test results that could impact their health, particularly when they have expressed interest in receiving such information. Furthermore, the return of genetic test results is essential to improve trust, transparency, and health equity. However, investigators often encounter barriers in returning genetic test results to research participants. We examined genomic research at a large, research-intensive medical school and found less than 6% of protocols included plans to return results to participants. This study describes our development of protocols for returning primary and secondary genetic test results and implementation of a Genomic Return of Results (gROR) service. This arose through a collaboration with experts in community engagement, genetics, and pathology to consider consent adequacy, analytical/clinical validity, and clinical utility when returning results. The gROR service reduces investigator burden and provides participants with genetic information and guidance to address any potential health risks. Genetic results are returned by a genetic counselor at no cost to participants or their family. Investigator costs are subsidized to incentivize the delivery of actionable genetic test results to research participants. Our approach prioritizes transparency, accessibility, and informed decision-making, thereby promoting equitable sharing of genetic knowledge and personalized healthcare interventions.

## Introduction

Efforts to increase transparency and access to research results are underway to include participants more fully as partners in research [1,2]. Participants consistently request their research results; researchers have a duty to respect participant autonomy and ensure they can access their results, when feasible. Transparency between researchers and participants promotes trust, enhances study recruitment and retention, increases subject diversity, and elevates the quality of research.

There are challenges associated with the return of any type of research results, including standard laboratory and imaging tests, and additional barriers for the return of genetic data, including determination of clinical validity and utility, participant consent, and burdens associated with cost and feasibility. The Precision Health function of the Washington University Institute of Clinical and Translational Sciences (ICTS) is funded by a National Center for Advancing Translational Science (NCATS) Clinical and Translational Science Award (CTSA), which aims to improve the efficiency, quality, and impact of research for improving human health. We sought to build a collaborative structure comprising our institution/university and our partner institutions across urban and rural areas of Missouri and Illinois to increase research participant access to both primary and secondary genetic research results. Primary results are defined as genetic results directly related to the research study, while secondary results, or incidental findings, are defined as positive findings unrelated to the primary objective of the study [3]. The secondary results most likely to be returned to participants include those related to established genetic disorders, particularly when effective interventions are available to prevent or significantly reduce morbidity and mortality [4]. The American College of Medical Genetics and Genomics (ACMG) actively curates a list of 84 clinically actionable disease genes, such as genetic risk factors for breast cancer and high cholesterol, that is often considered the standard set of secondary genes for which results should be returned [5].

While data sharing is required by many funding bodies, this only applies to the submission of deidentified data into repositories that are accessible by other investigators; there is currently no requirement that data, including genetic test results, are shared with participants. The ACMG recommends that clinical studies utilizing exome and genome sequencing return primary and secondary results from their selected curated gene list to patients [3], and it has been suggested that this should be similarly applied to research studies [6]. As the prevalence of reportable pathogenic and likely pathogenic variants depends on the aim and scope of each study, limited data is available regarding the frequency of returning primary results to research participants. Conversely, the prevalence of reportable pathogenic and likely pathogenic variants among the defined ACMG recommended list of secondary genes, which has increased over time and will likely expand in the future, ranges from 1%–3% [7–13]. While individuals with positive secondary results may not currently experience associated disease symptoms and may never develop the disease, they have a statistically increased risk of developing symptoms, often later in life. Despite this, it is unclear how often secondary research results are returned to participants. Many studies indicate that participants want to obtain their research results, and ethical arguments support the return of genetic results to participants, however, administrative, logistical, and cost barriers limit the return of genetic test results to participants [2,14–16].

To address this, our Precision Health function established a working group to develop recommendations and guidance for research teams on the return of genetic test results throughout all stages of their research process, from conception to implementation. This paper presents a comprehensive overview of the Genomic Return of Results service (gROR) that was created to implement these recommendations, and was built on the framework and model described by Darnell et al nearly a decade ago [6]. The workflow and guidelines that we present may guide other institutions in establishing similar services.

## Methods

### Needs assessment

To evaluate how investigators were returning genetic results in research studies at our institution, we worked with the Washington University Institutional Review Board (IRB) in August 2019 to identify all IRB-approved studies that collected biological samples from participants and reported in that the sample may be used for genetic or genomic research in the informed consent form, either at the time of the study or for future research. Next, the IRB asked investigators of studies that included a plan to return genetic or genomic results whether their IRB protocols and consents could be shared with us for evaluation of their approach to returning genetic results. We reviewed the protocols and characterized the details of the return of results plans for each study meeting these criteria.

### Service development

The goals and structure of the ICTS gROR service were developed over several years through a collaborative effort. A working group was convened through monthly meetings with key stakeholders, including members of ICTS Precision Health, IRB, and Human Research Protections Office (HRPO), representing genetic counselors, geneticists, neurologists, psychiatrists, pathologists, medical ethics consultants, and community engaged researchers. The ICTS Community Advisory Board was also consulted regarding the process and ethics of return of genetic results, particularly as it pertained to COVID-19 research participants. The main goal was to establish a guided pathway with built-in flexibility and oversight checkpoints for returning genetic research results to study participants. This gROR service also ensures that individual investigators, even those without a clinical background, have the expertise and resources to return genetic results.

### Consent review and guidance

The working group established guidelines for appropriate consent language for both data sharing and return of results to ensure that participants are adequately consented for return of genetic test results, then created a standardized genomics database consent form that was made available to investigators through the ICTS website (https://wustl.app.box.com/s/3dqhdprcnydvtvywxrcv909tgu1a4fz4). Investigators may utilize this standardized institutional consent form or create their own. Investigators who indicate their interest in return of results in their applications for IRB approval can easily contact the service for assistance with consent processes. Linking an existing IRB protocol to the gROR service protocol is simple for investigators with appropriate genetic consent in their protocols. To initiate gROR services, investigators enroll online, consult with the core, and receive workflow approval. They subsequently submit the approval documentation and a minor protocol revision to the IRB; no further IRB amendments are required.

### CLIA-compliant standards

Data returned to participants should be collected and stored under Clinical Laboratory Improvement Amendments (CLIA)-compliant guidelines. The gROR service recommends that investigators collect duplicate samples, especially if they are planning to perform sequencing and analyze samples in a non-CLIA research lab. At Washington University in St. Louis, we recommend storage of duplicate specimens in the WU Tissue Procurement Core (TPC), accredited by the College of American Pharmacists through the Biorepository Accreditation Program, (https://research.wustl.edu/core-facilities/tissue-procurement-core-siteman-cancer-center). The TPC recommends that secondary specimens be collected as either whole blood (in purple-top EDTA tubes) or buccal swabs (Oragene-Dx swabs [OCD-100, DNA Genotek, Ontario, Canada]) with two separate identifiers. Investigators are encouraged to include alternative procedures in their IRB approval and consent form language to facilitate flexible workflows.

### Data analysis and interpretation

Research studies generate genetic sequencing data from both internal and external sources. Depending on whether CLIA lab conditions were met during sample collection and sequencing, the gROR facilitates the collection of a second specimen, as well as variant validation and the generation of a CLIA lab report. Clinical testing is ordered by a member of the WashU Clinical Genetics team.

For secondary genetic findings, the gROR service collaborates with the Clinical Genomics Laboratory (CGL) in the Department of Pathology and Immunology at Washington University (https://pathology.wustl.edu/divisions/genomic-molecular-pathology/). A flat fee includes the following four services, with no extra cost for samples requiring variant validation: (1) collection of non-CLIA-certified genetic sequencing data from the investigator, (2) analysis of the DNA sequencing results to screen for ACMG secondary genetic results, (3) validation of any potential pathogenic or likely pathogenic variants in secondary genes in a CLIA-certified laboratory, and (4) generation of a clinical report. CGL quality assessment procedures ensure any discrepancies between analysis of initial research sequencing and CLIA-certified Sanger sequencing validation are evaluated and resolved. A typical report will contain the report date, results, clinical interpretation, and recommendations. These reports are returned directly to the genetic counselor in the gROR service.

### Genetic test report generation

A written report of the research genetic test result, conforming to the standards used to report genetic results to patients for clinical purposes, is required for a result to be returned to a research participant. Results that meet the following four criteria will be returned to the participant or family: (1) The result is analytically confirmed as established by sequencing or genotyping in a CLIA-certified laboratory or its equivalent (certified by another external body or agency). (2) The result is clinically confirmed, as determined by reasonable consensus among experts, and the reported result has a known causal association with disease in the tested population. Genetic variants classified as “pathogenic or likely pathogenic” meet these criteria. (3) The result has clinical utility and the participant can take beneficial action in response to the reported result. Pathogenic or likely pathogenic variants in ACMG genes meet these criteria. The “actionability” of genetic disorders is considered on a case by case by gROR leadership, as their actionability may be variable over time. (4) Investigators obtained informed consent from the participant, which includes a discussion of potential adverse ethical, legal, or social implications for the participant or third parties.

When the above four criteria for return of results are not met, a personalized action plan is created instead based on National Academy of Sciences recommendations [1]. Specifically, our gROR service considers the return of genetic variants on a case by case basis, recommending return of results when the value to a participant is high and the risks of harm are low and either (a) the reported results were not validated clinically, but rigorous sequencing or genotyping was performed, or (b) there is reasonable consensus among experts that the result has a known causal association with disease. For example, investigators may not be able to validate findings for deceased participants, or the ClinGen [17] gene-disease clinical validity may not be met, but the available literature supports causality. In these cases, a genetic counselor communicates the research results to the participant via an informal report containing information regarding the limits on test validity and interpretation, along with the resources to confirm the genetic results in the future. The informal report outcomes are not incorporated into the electronic health record. Investigators are not responsible for maintaining and storing samples and sequence data beyond what is required by their IRB protocols. Negative results will not be returned through our service, but may be communicated by the investigator if approved in their IRB.

### Genetic counseling

A genetic counselor or medical geneticist communicates the genetic test results documented in the CLIA-certified laboratory report to participants so that they can follow up with an appropriate specialist. The genetic counselor also provides a list of resources and guidance for further steps the participant can take to address health risks to them or their family. Genetic counseling services are subsidized by our center NIH CTSA award, but may also be performed as a clinical visit billed to insurance. The gROR service and investigators are not responsible for any additional laboratory tests or procedures that the participant may seek based on the genetic test report.

### Cost estimation for grant budgeting

The financial cost of gROR is the most important consideration for many investigators. These workflows should be designed, verified, and established before investigators write the budgets for their grant applications. The investigator is responsible for all applicable sample collection, sequencing, and variant validation costs. Some costs apply to all participants, while some apply only to participants with genetic variant data that will be returned. Our gROR service collaborates with the Tissue Procurement Core for sample storage ($10–30 per sample as needed). The CGL (Washington University Department of Pathology and Immunology) provides ACMG sequencing data analysis and validation at a fixed cost of $70 per sample, regardless of whether a variant is identified that will be returned to a participant. For other studies in which the data analysis is performed by the investigator, single variant validation in a CLIA laboratory costs $200–400 per variant. The cost of a genetic counselor visit is currently $400–500 and may be covered by the participant’s insurance.

The gROR service currently subsidizes investigator consultations, administrative assistance with obtaining samples and submitting them for CLIA validation, and genetic counseling services. Any follow-up specialist visits, laboratory tests, or procedures are not the responsibility of the ICTS or the research investigator.

## Results

### Institutional needs assessment of genomic return of results

**Figure 1.**
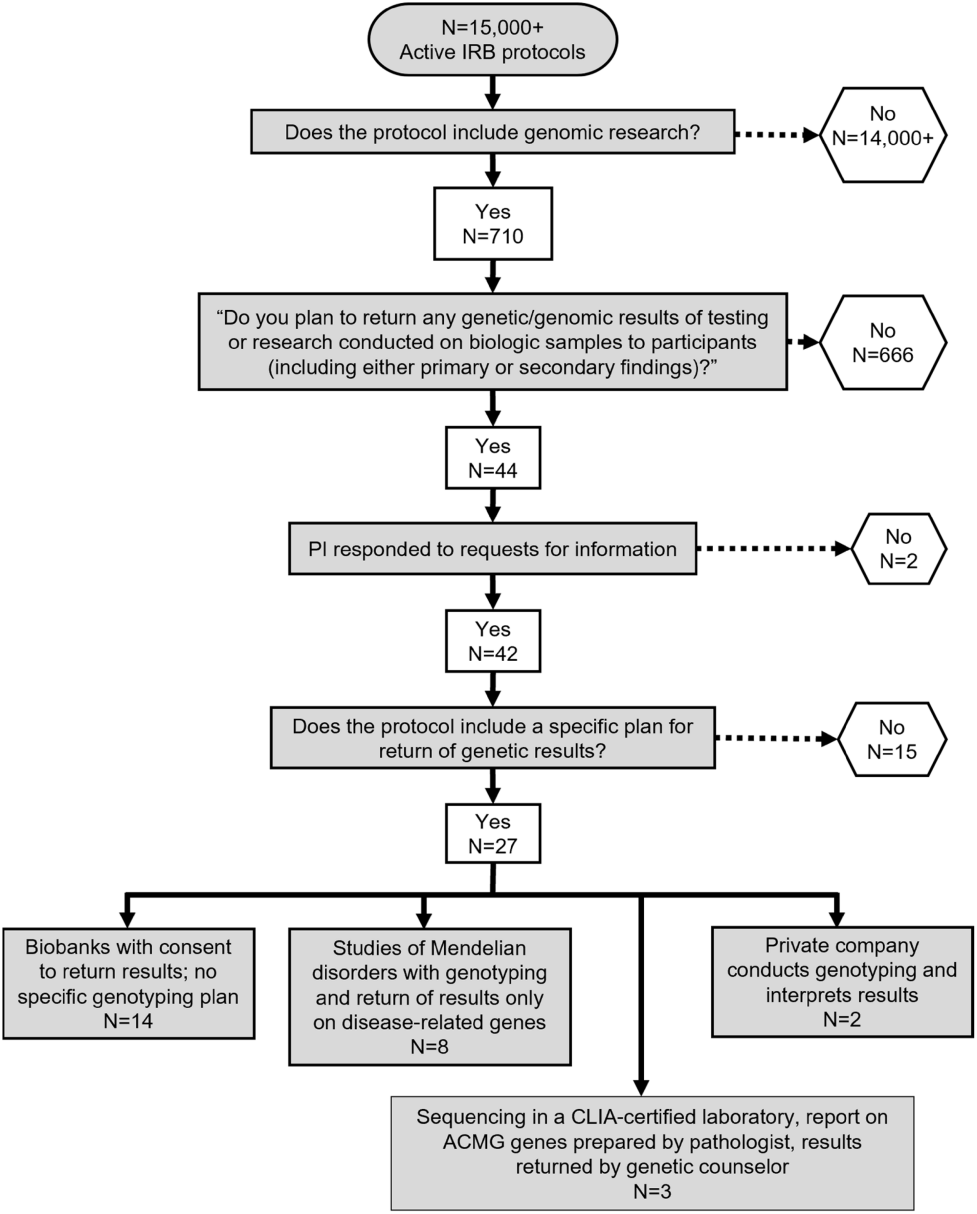
Needs assessment for return of genomic data. Shown are the total number of IRB protocols, the number that include genomic research, and those with plans to return results to research participants at Washington University School of Medicine in 2019. Only three studies planned to return secondary findings to participants.

Of the >7000 active IRB protocols at Washington University in August 2019, 710 protocols included genomic research, of which only 44 protocols (6%) reported an intention to return results to participants (Figure 1). We obtained permission from investigators to review 42 of these protocols for additional study details and found that only 3 protocols specified a plan to return secondary genetic findings from the ACMG genes.

### Genomic return of results service implementation

**Figure 2.**
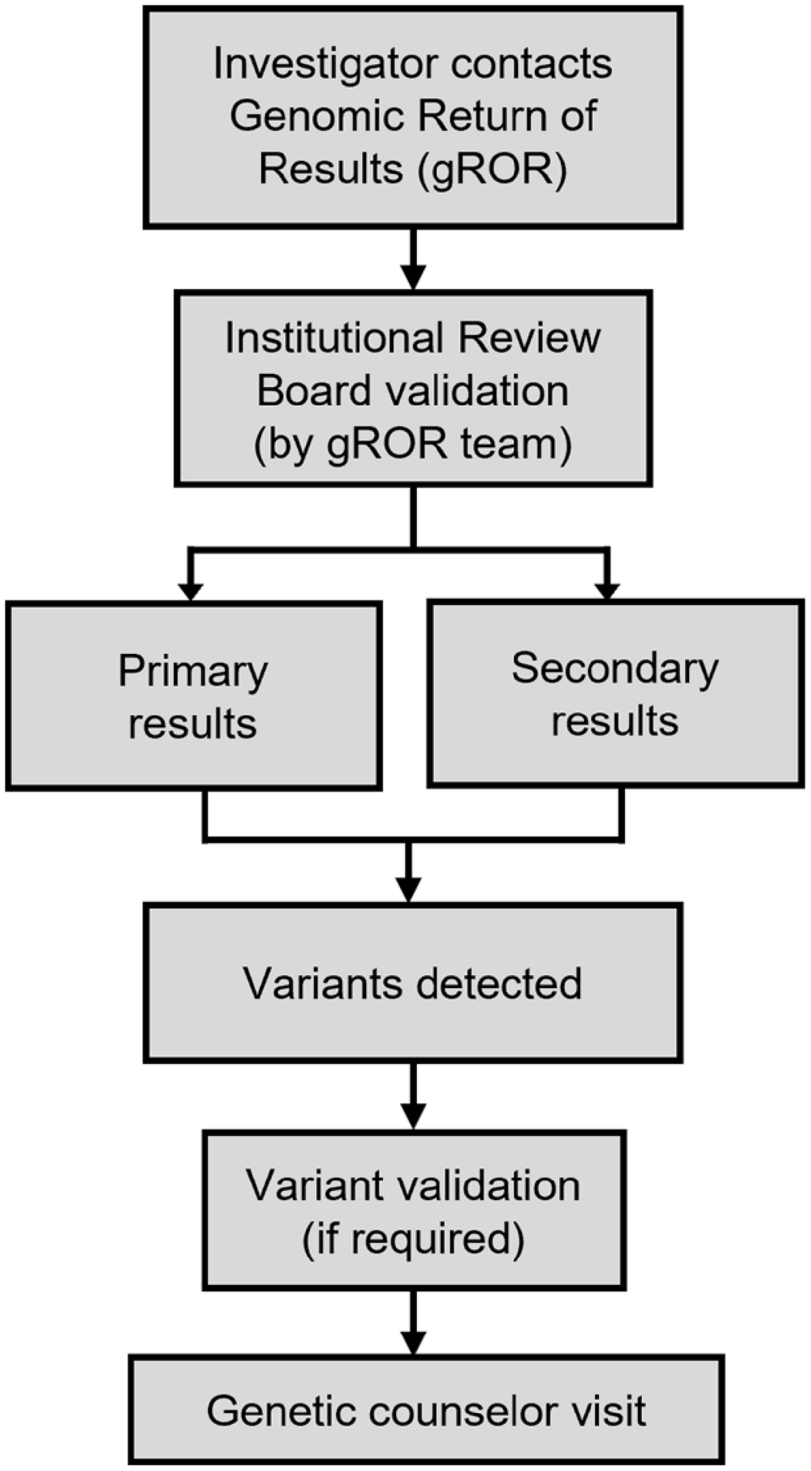
Overview of the ICTS Precision Health Genomic Return of Results (gROR) service core.

The gROR service was established in 2023 and implemented in 2024. The process for utilizing the gROR service is described in detail on our website (https://icts-precisionhealth.wustl.edu/genomic-tools/return-of-results; Figure 2). Accessing gROR services consists of five steps: (1) Investigators complete a service request. (2) Investigators consult with the gROR service to discuss their study needs. (3) Investigators provide additional specific study information. (4) The gROR service contacts HRPO/IRB to verify that the consent and return of results plans have been approved. (5) Investigators work directly with the gROR service to provide CLIA-compliant sample collection/storage, variant interpretation, and clinical genetics, as needed for the specific study. The investigator may be responsible for some costs incurred during this process, which can vary depending on the study requirements. In the first two years, seven investigators applied for the gROR service.

### Examples of gROR service usage: primary results

Primary genetic test results of a research study are individualized by the investigator based on the nature of the study but often include genes that are listed as disease genes in ClinVar. Here, we present an example of a primary genetic result that was returned to demonstrate the type of study and participant who have benefited from this new gROR service. A research study of autism identified the same pathogenic *de novo* variant in a new disease gene (*KCNC2*) in two affected siblings using non-CLIA research sequencing. A case study describing these two siblings was published [18], and the results were deemed significant to the siblings’ health because they ultimately impacted the treatment of their epilepsy [19]. The investigator initially requested reanalysis of exome sequence data that had been acquired by a commercial vendor during routine clinical care. However, the commercial laboratory would not report the variant because the gene did not yet have ClinGen-curated gene-disease clinical validity. Therefore, the investigator completed a consultation with the gROR, and the results met the service criteria. The treating physician informed the siblings’ parents that a genetic variant was identified that could explain their autism. The gROR service facilitated the CLIA-compliant collection of samples that were sent to a commercial laboratory [GeneDx (Gaithersburg, MD)] for Sanger validation, which was confirmed and documented in a clinical report generated by GeneDx. The investigator’s research project covered the cost of this genetic confirmation. A genetic counselor returned the clinical report describing the variant to the parents during a clinical genetics visit.

### Examples of gROR service usage: Secondary results

**Figure 3.**
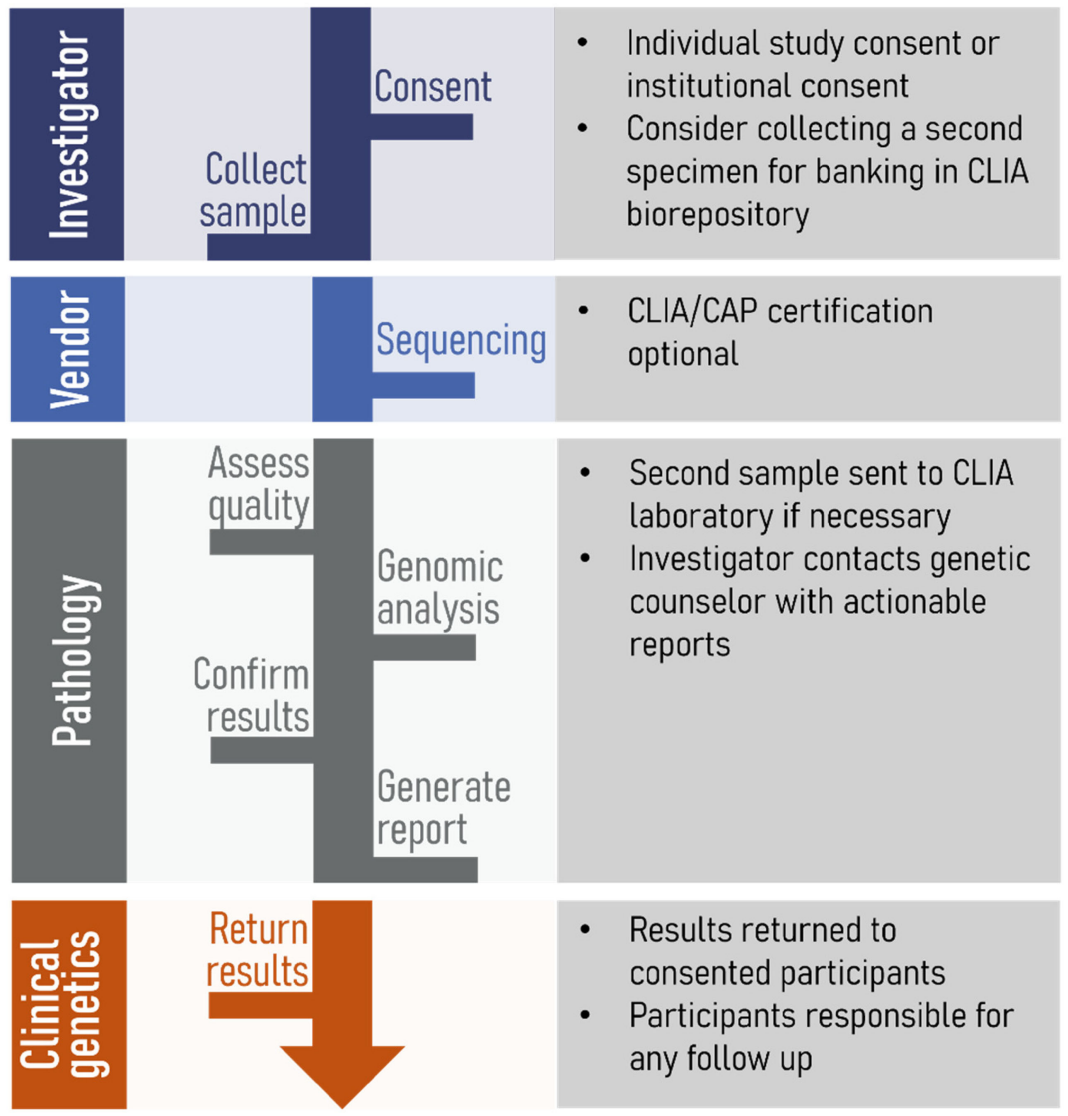
Overview of the steps involved in the return of secondary genetic results. The Genomic Return of Results service helps to coordinate actions by the investigator, sequencing vendor, pathology/data analytics, and clinical genetics. CLIA = Clinical Laboratory Improvement Amendments; CAP = College of American Pathologists.

Our service provides investigators with guidance on the return of secondary genetic results (Figure 3). The gROR service offers customized guidance for secondary results depending on where the samples were sequenced and analyzed. For example, gene variants may be discovered during analysis in an investigator’s laboratory, a commercial laboratory, or an institutional core.

Here, we present a case of secondary genetic results that were returned through the gROR service. A five-year-old adopted child’s DNA was exome sequenced as part of a research study to identify the causes of clubfoot. The results identified a pathogenic variant in a clinically actionable disease gene associated with aortic aneurysm. Unfortunately, the child died suddenly and unexpectedly of unknown cause several months before the pathogenic gene variant was identified. As the variant may have contributed to the participant’s death, the gROR expert committee was convened and determined that it was important to return this result to the child’s adopted family, even though another sample could not be obtained for CLIA validation. A geneticist and the clinician involved in the patient’s care returned the results to the participant’s adoptive parents. The patient’s biological parents were not contactable.

## Discussion

Our needs assessment demonstrated that many investigators conduct genomic research and have stated an interest in returning results to participants. However, less than 6% of genomic research studies at our institution reported an intention to return the results and less than 1% included specific plans to return the recommended secondary genetic findings, highlighting the need for additional support. Despite the publication of a framework and model for returning secondary genomic findings nearly 10 years ago [6], implementation of such services has been patchy. While the current utilization of gROR is poor nationally and often limited to research studies of its implementation [20], recent literature provides an increasing number of examples of its application [21–25], including international efforts and the large-scale efforts of the All of Us research program [26,27]. Even large scale efforts encounter challenges in returning research results, with the All of Us research program recently pivoting from offering return of research results to offering no-cost clinical testing of select genes outsourced to a clinical company [28]. This emphasizes the importance of our work, as this alternate strategy deployed by All of Us is duplicative, yet may be the most efficient, and perhaps cost effective, solution.

Our gROR service offers process simplification and guidance to all stakeholders for every stage of the process, from the initial consent and protocol development to the actual return of results to participants, thereby reducing labor and cost for the investigative team. The fundamental principles of the Belmont Report, such as justice, beneficence, and autonomy, and the ethical concept of “duty to rescue” are applicable to our shared efforts to return genetic data to research participants, similar to other clinical service examples [6,25,29,30]. Our gROR service utilization has grown from only one consultation in the first year to six in the second year, collectively representing more than 300 research participants. While most applicants to the gROR service were individual researchers, one request was related to a University of Missouri-Columbia biorepository, which now requires mandatory return of secondary findings through our service for any tissues used for other research studies. Many consultations have been to support the development of studies with planned future return of results. Currently, investigators who engage with our service are seeking to return of an even mix of primary, secondary, or both primary and secondary results. All investigators have informed us that without the service they would not be able to provide results back to the study participants. In the first two years, results were returned to seven participants.

Despite establishing gROR service at our institution, we identified additional barriers to its widespread use: (1) Users are limited to faculty members of Washington University ICTS or an ICTS partner institution. Establishing a national service would be helpful, but sharing genomic data makes this unlikely. (2) Despite subsidization by the institution, use of the gROR service includes financial costs to investigators. Costs are unpredictable over time due to the changing academic landscape, which is challenging for investigators. (3) Marketing and awareness of the gROR service in a diverse community of research investigators within a siloed academic environment is challenging. Our current IRB process asks specifically about return of results during protocol development, which has improved awareness and referrals. Future work will continue to refine the workflow processes and build our cohort of investigator users. As investigators obtain funded grant awards that include the cost of the service, program stability and sustainability will be ensured and the above barriers may be mitigated.

Since launching our gROR service core, referrals from other institutions within the CTSA consortium have increased, highlighting the need for such services. Sharing our service structure and outcomes with IRB committees has enhanced the credibility and utility of our gROR service, helping other institutions consider similar services. Our efforts emphasize participant-centered practices in genomic research, promoting a standardized and ethical approach to returning genetic results.

## Conclusions

Returning genetic results to research participants requires meticulous planning by the research team. The gROR service streamlines this process, offering support at every stage and aiding in the efficient and cost-effective return of results to consenting participants. This paper outlines the structure and methods for investigators to return genetic research results, emphasizing the importance of innovative solutions, simplified processes, and ongoing monitoring due to evolving landscapes. Key insights include the importance of institutional cooperation, individualization through consultation, clear communication, efficiencies in centralization of the process, and resource investment for successful implementation.
